# Hematological change parameters in patients with pressure ulcer at long-term care hospital

**DOI:** 10.1590/S1679-45082014AO3034

**Published:** 2014

**Authors:** Giselle Protta Neiva, Julia Romualdo Carnevalli, Rodrigo Lessa Cataldi, Denise Mendes Furtado, Rodrigo Luiz Fabri, Pâmela Souza Silva

**Affiliations:** 1 Faculdade de Ciências Médicas e da Saúde de Juiz de Fora, Juiz de Fora, MG, Brazil.; 2 Prefeitura Municipal de Juiz de Fora, Juiz de Fora, MG, Brazil.; 3 Universidade Federal de Juiz de Fora, Governador Valadares, MG, Brazil.

**Keywords:** Pressure ulcer/drug therapy, Pressure ulcer/prevention & control, Wound healing, Hematocrit, Hemoglobins

## Abstract

**Objective:**

To assess factors associated with the development of pressure ulcers, and to compare the effectiveness of pharmacological treatments.

**Methods:**

The factors associated with the development of pressure ulcers were compared in lesion-carrying patients (n=14) and non-carriers (n=16). Lesion-carrying patients were treated with 1% silver sulfadiazine or 0.6IU/g collagenase and were observed for 8 weeks. The data collected was analyzed with p<0.05 being statistically relevant.

**Results:**

The prevalence of pressure ulcers was about 6%. The comparison of carrier and non-carrier groups of pressure ulcers revealed no statistically significant difference in its occurrence with respect to age, sex, skin color, mobility, or the use of diapers. However, levels of hemoglobin, hematocrit, and red blood cells were found to be statistically different between groups, being lower in lesion-carrying patients. There was no significant difference found in lesion area between patients treated with collagenase or silver sulfadiazine, although both groups showed an overall reduction in lesion area through the treatment course.

**Conclusion:**

Hematologic parameters showed a statistically significant difference between the two groups. Regarding the treatment of ulcers, there was no difference in the area of the lesion found between the groups treated with collagenase and silver sulfadiazine.

## INTRODUCTION

Pressure ulcers (PU) are a severe health problem worldwide. In Brazil, few studies refer to the incidence and the prevalence of ulcers, although the problem is similar to what is found in other countries, with an incidence of 2 to 29.5% in the general population.^(1)^ Moreover, in long-term care stay hospitals, the scenario is even more concerning, given roughly 40% of the elderly hospitalized develop ulcers during their inpatient stay.^(2)^ Although very frequent, the difficulties and challenges for prevention and treatment have been discussed for many years.^(2)^

A PU can be defined as a localized lesion, affecting skin and/or subjacent tissues, usually on a bone prominence, resulting from pressure or pressure associated with shear force and/or friction.^(3)^ Depending on the tissue affected, the development of an ulcer is classified into stages (I to IV), based on the criteria of the National Pressure Ulcer Advisory Panel (NPUAP).^(3)-5)^ Stage I of the ulcer is characterized as observable changes on intact skin, with persistent erythema of a localized area and that does not disappear upon pressure, in addition to variations in tissue temperature (warm or cold), when compared to adjacent tissue. Additionally, the area may be painful, hardened or softened. Stage II ulcers are characterized by partial loss of width of the derma, presenting as a superficial ulcer with a pale red color bed, without slough. They can also present as an intact or open phlyctena. Stage III ulcers, conversely, are characterized by loss of total thickness of tissue. Subcutaneous fat may be visible, without exposure of the bone, tendon or muscle. At this stage, the slough may be present, without compromising the identification of the depth of the tissue loss. Last, the total tissue loss with exposure of the bone, muscle or tendon is seen on stage IV ulcers. In addition, there may the presence of slough or an eschar on some parts of the wound bed.

The etiology of PU is due to extrinsic and intrinsic patient factors. Among extrinsic factors, the main ones include intensity and duration of the agent that presses the skin, while, among the intrinsic ones, patient age, nutritional status and level of conscience, tissue perfusion, use of some medications, and the presence of chronic conditions, such as diabetes and cardiovascular diseases, stand out.^(4)^

Initial care for treatment of an ulcer may involve debridement, with posterior application of cleansing and tissue regeneration products.^(5)^ Saline at 0.9% is frequently used for cleaning, followed by several pharmacological agents, such as essential fatty acids, collagenase and silver sulfadiazine.^(6)^

In Brazil, there are few ongoing studies on PU and their treatment. Studies performed show that forms of treatment vary greatly, in addition to showing that existing guidelines are sometimes inappropriately followed.^(2, 7)^

## OBJECTIVE

To evaluate factors related to the development of pressure ulcers in inpatients at a long-term care stay hospital, and to evaluate how to compare the effectiveness of two pharmacological treatments**.**

## METHODS

The present study is a prospective study, performed on inpatients at a long-term care stay hospital with 219 beds in the city of Juiz de Fora (MG). The study included patients with PU (n=14) admitted to the institution during the period assessed, from March to May 2013, and patients at risk for developing ulcers (n=16), which were selected randomly based on the Braden scale <18. Such scale is a useful tool and used frequently to assess the risk for developing PU, determined through the measurement of clinical exposure parameters to intense and prolonged pressure, and of tissue tolerance to this pressure. The Braden scale score ranges from 4 to 23, in that inpatients with a score ≥16 are considered of low risk for developing lesions, while scores <11 point toward a high risk.

The present study was approved by the Ethics in Research Committee of *Faculdade de Ciências Médicas e da Saúde de Juiz de Fora* (process 0059/12), and all volunteers received information on the study and consented to take part by signing the Consent Form.

Data were collected from patient charts, aimed at registering information on socio-demographic, clinical, systemic medication, and risk factors, measures to prevent ulcers, and follow-up of pharmacological and non-pharmacological treatment of lesions. Data on lab tests (hemoglobin, hematocrit, red blood cells, leucocytes, neutrophils, lymphocytes, glucose, creatinine, total cholesterol and fractions and triglycerides) were obtained from blood samples of appropriately fasting patients, collected after their inclusion in the study. All tests were performed according to hospital routine. The pharmacological treatment of lesions was chosen between 1% silver sulfadiazine (dermatologic cream) (n=6) and 0.6UI/g collagenase (pomade) (n=8), according to the attending physician and without interfering in the routine of the institution.

All treatments were applied once a day, in the morning, after a trained professional cleaned the wound with saline. The wound remained covered with gauze during the entire treatment.

After beginning the assessment, patients were followed during 8 weeks, with 15 day intervals, totaling 4 assessments. Parameters regarding the wound (size, aspect and pain) were considered in each assessment, and recorded in a form developed for the study. Measurements of size of lesions took into account the length (largest longitudinal axis, border to border of the lesion bed) and width (smallest axis transversal to longitudinal axis, border to border of the lesion bed), using a pachymeter and recorded in centimeters. The total area of the wound was calculated by multiplying length by width (cm^2^). In addition to pharmacological treatments, non-pharmacological measures performed also were recorded for posterior comparison between patient groups.

Data collected were processed and analyzed by the GraphPad Prism, version 5.0 program. After tabulation, data were submitted to the following statistical procedures: Fisher’s exact test to compare nominal variables such as sex, color, mobility and wearing a diaper between the group of patients with PU and those without a wound; and χ^2^ test to compare groups in relation to disease prevalence. The difference between groups in relation to total medications being used was done using χ^2^ test, while the comparison between each one of the classes of medication was done using Fisher’s exact test. All continuous variables were evaluated as to normal distribution (D’Agostino and Pearson test). For those who presented normal distribution, groups were compared by non-paired *t* test analysis, while data without normal distribution were compared by the non-parametric Mann-Whitney test.

All analyses used p<0.05 as statistically significant.

## RESULTS

Among the 219 beds available at the long-term care stay hospital in the city of Juiz de Fora, 14 patients with PU were identified during the 3 months on which the study was conducted, showing the prevalence of roughly 6% for this type of wound. Among such patients, two died due to sepsis and one was transferred to another health institution during the observation period.

In addition to patients with ulcers, patients at risk for developing a lesion also were assessed, and such general characteristics were compared to those patients with lesions (Table 1). The comparison between both groups showed there was no statistically significant difference in the occurrence of pressure ulcers, according to age, sex, color of skin, mobility and wearing diapers. However, when hematological and biochemical parameters were compared (Table 2), differences in hemoglobin, hematocrit and red blood cell parameters were observed, in that such values were low in patients with wounds. On the other hand, significant differences were not observed when comparing different classes of medications used by groups (Table 3).

**Table 1:** General characteristics of inpatients (n=30) with pressure ulcers or at risk for developing a lesion at a long-term care stay hospital

Characteristic	With ulcer (n=14)	Without ulcer (n=16)	p value
Age	63.6±15.4	64.25±17.67	0.92
Sex			
Male	7	9	
Female	7	7	1.00
Color			
White	8	5	
Not white	6	11	0.27
BMI (kg/m^2^)	20.4±2.5	20.7±2.8	0.70
Nutritional status			
Malnutrition	3	2	
Eutrophy	8	13	
Overweight	0	1	0.47
Mobility			
Walks	3	0	
Does not walk	11	16	0.09
Conditions			
Neurologic conditions	10	13	
LLLL Lesions	5	3	
Diabetes	2	3	
HAS	7	7	
Urinary infection	4	0	
Others	2	6	0.22
Wearing diaper			
Yes	12	15	
No	2	1	0.60
Use of “egg box” mattress			
Yes	8	1	
No	6	15	-
Number of ulcers per patient	2 (Minimum: 1/Maximum: 5)*	-	-

Results expressed by mean ± standard deviation.

(-) Statistical analysis not performed; * amplitude of occurrence of pressure ulcers per patient.

BMI: body mass index; LLLL: lower limbs; HAS: systemic arterial hypertension.

**Table 2:** Biochemical and hematological characterization of inpatients (n=30) with pressure ulcers or at risk for developing a lesion at a long-term care stay hospital

Feature	With ulcer (n=14)	Without ulcer (n=16)	p value
Hemoglobin (g/dL)	10.5±1.3	12.8±1.5	0.0002*
Hematocrit (%)	32.6±3.9	39.9±4.3	<0.0001*
Red blood cells (millions/mm^3^)	3.9±0.6	4.7±0.7	0.0026*
Leucocytes (n/mm^3^)	5.877±1.562	6.694±1.750	0.2009
Neutrophils (%)	60.4±10.8	67.6±11.7	0.1008
Lymphocytes (%)	32.5±10.4	26.5±10.0	0.1232
Glucose (mg/dL)	80±13	86±20	0.3984
Creatinine (mg/dL)	0.8±0.4	0.8±0.3	0.8134
Total Cholesterol (mg/dL)	174±27	207±50	0.0631
LDL (mg/dL)	111±22	138±48	0.1025
HDL (mg/dL)	37±13	36±7	0.6959
Triglycerides (mg/dL)	135±76	159±109	0.8330

Results expressed by mean ± standard deviation.

* p<0.05 (non-paired t test).

LDL: low- density lipoprotein; HDL: high density lipoprotein.

**Table 3:** Prevalence of pressure ulcers according to systemic medication being used by patients

Medication	With ulcer (n=14)	Without ulcer (n=16)	p value
Anemia medication	19 (22)	18 (18)	0.596^3a^
Antibiotic	3 (3)	2 (2)	1.0000^a^
Psychotropic	21 (24)	19 (19)	0.4915^a^
Insulin and oral hypoglycemic	1 (1)	7 (7)	0.0649^a^
Anti-hypertensive	9 (10)	9 (9)	1.0000^a^
Analgesic	4 (5)	4 (4)	1.0000^a^
Diuretic	6 (7)	10 (10)	0.6133^a^
Others	23 (27)	30 (30)	0.7542^a^

Total	86 (100)	99 (100)	0.479^1b^

^a ^Fisher´s exact Test

^b ^χ^2^Test.

For patients with PU, location and classification of wounds are shown on table 4, highlighting the predominance of ulcers on the sacral region, followed by the calcaneus and trochanter region.

**Table 4:** Characteristics of pressure ulcers in 14 inpatients at a long-term care stay hospital

Characteristic	Number of ulcers (n=26) n (%)
Location	
Sacral	12 (46)
Trochanter	2 (8)
Calcaneus	7 (27)
Others	5 (19)
Stage	
I	3 (12)
II	9 (34)
III	11 (42)
IV	3 (12)

**Figure 1:**
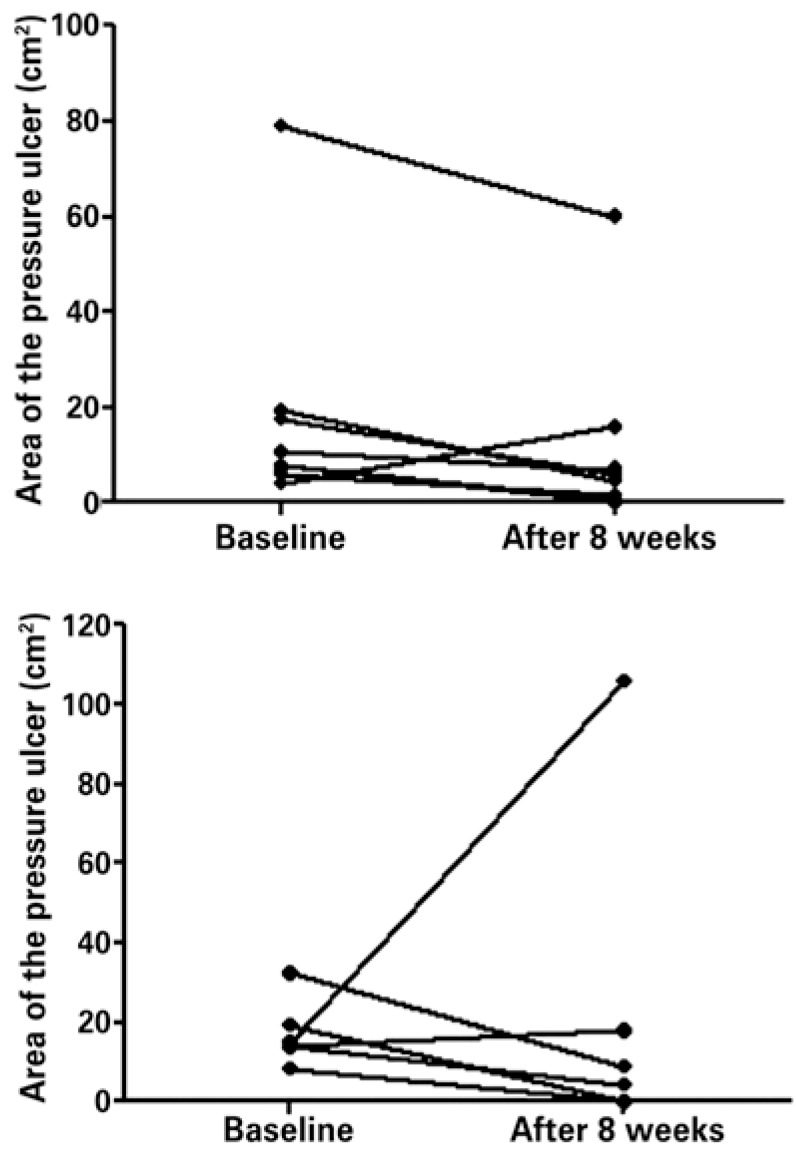
Measurements of pressure ulcers (cm2) in 11 inpatients at a long-term care stay hospital before and after treatment (A) collagenase (n=8) and (B) sulfadiazine (n=6) for 8 weeks. No significant differences were observed in baseline values and those after treatment during the follow-up period (p>0.05)

The development of the ulcer in relation to treatment (collagenase or silver sulfadiazine) was monitored using the lesion area, during the assessment period of 8 weeks (Figure 1). Although most patients had a reduction in the average size of the wound, the comparison of the lesion area before and after pharmacological treatment did not show a statistical difference. Still related to the reduction of the lesion area, a significant difference between both pharmacological treatments (collagenase or silver sulfadiazine; p=0.94) was not observed.

## DISCUSSION

The data presented in this study showed that the prevalence of pressure ulcers found at the hospital investigated in the study (about 6%) are in agreement with the international literature that shows a range between 3 and 14%, in the United States. In contrast, studies in Brazil state that the prevalence at hospitals can reach approximately 20%.^(4, 8)^

In addition to patients with PU, during the same period, a group of patients at risk for developing the lesion was also assessed. Such comparison showed no significant statistical difference regarding age, sex, mobility and wearing diapers. Regarding color of skin, there also was no significant predominance between groups (p=0.27), unlike previous observations suggesting that black skin is more resistant to external aggression caused by humidity and friction, due to changes in the corneal layer, which is more compact in black individuals.^(4)^ Additionally, our data did not show a statistically significant difference between the body mass index (BMI) and the nutritional level of patients with and without lesions, unlike previous findings that showed a direct relationship between BMI and the prevalence of ulcer growing in malnutrition, due to the loss of the “buffer effect” of fat tissue.^(9)^ Such discordance in results may be due to the fact that both groups of patients investigated in the present study showed a BMI near the lower limit of normality, as most patients were bedridden for a long period because of their baseline conditions. Moreover, another possible explanation for the discordance among data presented in the present study and those presented in the literature may be the different methods used for assessing patient nutritional status.^(9)^

On the other hand, regarding biochemical and hematological data analyzed between patients who developed pressure ulcers and patients without ulcers, there was a significant reduction in hemoglobin (p=0.0002), hematocrit (p<0.0001) and red blood cells (p=0.026) values in patients with lesions. Several diseases are known to be possibly associated with the development of pressure ulcers, such as *diabetes mellitus*, hypertension, neurological conditions and infections, but only anemia showed a significant relationship in our study.^(9)^ Red blood cell deficiency is common in patients with pressure ulcers, not only due to deficient nutritional status, but also due to the effects of inflammatory cytokines, consequent to the lesion, on erythroid progenitor cells.^(10)^ These conditions limit patient mobility and compromise blood irrigation, reducing transportation of oxygen and of nutrients to the wound.^(9)^

Conversely, no statistically significant association was observed between the value of total leucocytes (p=0.2009) and the development of a pressure ulcer, comparing the two groups observed, and likewise for neutrophils (p=0.1008). According to the literature, the increase in neutrophils seems to indicate a bad prognosis of pressure ulcers, due to inflammatory mediators triggered by lesions, also raising total leucocytes.^(9)^ However, in the present work, this association was not observed.

When evaluating medications being used by inpatients, there was a great diversification of classes, with predominance of psychotropics, followed by anemia medication and anti-hypertensives, without a statistically significant difference in the use by patients in groups with and without lesions (p=0.4791). Regarding medications used, it is known that especially those on continuous use, although necessary, may contribute to the development of an ulcer, such as hypotensive agents, that may change blood flow, reducing tissue perfusion and leading to skin tolerance to pressure.^(4)^

As to location of pressure ulcers, data found are in agreement with what is described in the literature, presenting a predominance of ulcers in the sacral region, followed by the calcaneus and trochanter region.^(4, 11)-15)^ Such fact is explained by the dorsal horizontal position, that potentiates friction and shear forces on patients that remain for a long time in the same position.^(11)^

The comparison of the area of the wound between the beginning and end of the 8-week observation time showed there was no significant difference as to reduction after both treatments. However, these values were very near the limit established of p=0.05, given this comparison reached a value of p=0.06 for the treatment with collagenase, and the p value was 0.07 for silver sulfadiazine (excluded from this last analysis a patient with clinical complications because of a recurrent urinary infection, which probably explains the growth of the area of the sacral wound after treatment). Moreover, the comparison between both pharmacological treatments did not show a difference as to the reduction in the lesion area during the 8 weeks of observation.

Despite interesting findings, the reduced number of patients and the short observation period of pharmacological treatments (8 weeks) were an important limitation of the study.

## CONCLUSION

The prevalence of pressure ulcers at a long-term care stay hospital in Juiz de Fora shows agreement with domestic and international data on the theme.

Additionally, a significant statistical relation was observed between the development of pressure ulcer and low hemoglobin, hematocrit and red blood cell values, and these parameters may be useful to identify patients at risk for developing a lesion, in addition to the monitoring and treatment of patients with this wound. In this sense, such findings, added to measures already known for preventing pressure ulcers, become extremely relevant information to be amply disclosed and incorporated into the routine of health professionals who work with patients at risk for developing a wound.

At the end of the assessment, the comparison between treating pressure ulcers with collagenase or silver sulfadiazine showed no significant statistical difference in the decrease in the wound area, although both collagenase and sulfadiazine showed a mean reduction in the lesion.
