# Supplementary material for: Hematological change parameters in patients with pressure ulcer at long-term care hospital
Source: Einstein (Sao Paulo). 2014 Jul-Sep;12(3):304–9. doi: 10.1590/S1679-45082014AO3034 (PMC4872940; doi:10.1590/S1679-45082014AO3034)
Supplement: Translation

# Translation: Alterações dos parâmetros hematológicos em pacientes portadores de úlcera por pressão em um hospital de longa permanência

**Authors:** Giselle Protta Neiva, Julia Romualdo Carnevalli, Rodrigo Lessa Cataldi, Denise Mendes Furtado, Rodrigo Luiz Fabri, Pâmela Souza Silva

**Affiliations:** 1 Faculdade de Ciências Médicas e da Saúde de Juiz de Fora, Juiz de Fora, MG, Brasil.; 2 Prefeitura Municipal de Juiz de Fora, Juiz de Fora, MG, Brasil.; 3 Universidade Federal de Juiz de Fora, Governador Valadares, MG, Brasil.

**Keywords:** Úlcera por pressão/quimioterapia, Úlcera por pressão/prevenção & controle, Cicatrização, Hematócrito, Hemoglobinas

## Abstract

**Objetivo:**

Avaliar os fatores associados ao desenvolvimento da úlcera por pressão e comparar a efetividade de tratamentos farmacológicos.

**Métodos:**

Os fatores associados ao desenvolvimento de úlcera por pressão foram comparados entre pacientes portadores (n=14) e não portadores (n=16) da lesão. Os pacientes com a ferida foram tratados com sulfadiazina de prata 1% ou colagenase 0,6UI/g, sendo acompanhados durante 8 semanas. Os dados coletados foram analisados considerando-se estatisticamente significativo se p<0,05.

**Resultados:**

A prevalência de úlcera por pressão foi de cerca de 6%. A comparação entre os grupos de portadores e não portadores de úlcera por pressão não revelou diferença estatística significativa entre ocorrência desta segundo idade, sexo, cor da pele, mobilidade e uso de fralda. Por outro lado, os valores de hemoglobina, hematócrito e hemácias foram estatisticamente diferentes entre os grupos, sendo menor naqueles pacientes portadores da lesão. Não houve diferença significativa na área da lesão entre aqueles pacientes tratados com colagenase ou sulfadiazina de prata, embora ambos os tratamentos tenham evidenciado uma redução média da área da lesão.

**Conclusão:**

Os parâmetros hematológicos apresentaram diferença estatística entre os dois grupos avaliados, ao contrário das demais variáveis analisadas. Em relação ao tratamento das úlceras, não houve diferença na área da lesão na comparação entre a colagenase e a sulfadiazina.

## INTRODUÇÃO

A úlcera por pressão (UP) é um grave problema de saúde em todo o mundo. No Brasil, poucos são os estudos referentes à incidência e à prevalência de úlceras, contudo, o problema é semelhante ao encontrado em outros países, apresentando incidência de 2 a 29,5% na população em geral.^(1)^ No entanto, em hospitais de longa permanência, o cenário é ainda mais preocupante, já que cerca de 40% dos idosos internados desenvolvem úlcera durante seu período de internação.^(2)^ Apesar de muito frequente, as dificuldades e os desafios da prevenção e tratamento são discutidos há muitos anos.^(2)^

A UP pode ser definida como uma lesão localizada, acometendo pele e/ou tecidos subjacentes, usualmente sobre uma proeminência óssea, resultante de pressão ou de pressão associada a cisalhamento e/ou fricção.^(3)^ De acordo com o comprometimento tecidual, a evolução da úlcera é classificada em estágios (I a IV), tendo por base os critérios da *National Pressure Ulcer Advisory Panel* (NPUAP).^(3)-5)^ O estágio I da úlcera caracteriza-se por alterações observáveis na pele intacta, com eritema persistente de uma área localizada e que não desaparece a pressão, além de variações da temperatura tecidual (quente ou fria), quando comparada com o tecido adjacente. Adicionalmente, a área pode se apresentar dolorosa, endurecida ou amolecida. Por outro lado, as úlceras no estágio II são caracterizadas pela perda parcial da espessura da derme, apresentando-se como uma úlcera superficial com o leito de coloração vermelho pálida, sem esfacelo. Pode ainda se apresentar como flictena, intacta ou aberta. Já as úlceras em estágio III são caracterizadas pela perda de tecido em sua espessura total. A gordura subcutânea pode estar visível, sem exposição do osso, tendão ou músculo. Nesse estágio, o esfacelo pode estar presente, sem prejudicar a identificação da profundidade da perda tecidual. Por fim, a perda total de tecido com exposição do osso, do músculo ou do tendão é observada nas úlceras em estágio IV. Em adição, pode haver presença de esfacelo ou escara em algumas partes do leito da ferida.

A etiologia da UP deve-se a fatores extrínsecos e intrínsecos ao paciente. Dentre os fatores extrínsecos, os principais incluem a intensidade e a duração do agente que pressiona a pele, enquanto, dentre os intrínsecos, destacam-se a idade do paciente, seu estado nutricional e nível de consciência, a perfusão tecidual, o uso de alguns medicamentos e a presença de doenças crônicas, como o diabetes e doenças cardiovasculares.^(4)^

O cuidado inicial para tratamento da úlcera pode envolver o desbridamento, com a posterior aplicação de produtos para limpeza e regeneração tecidual.^(5)^ A limpeza é frequentemente realizada com soro fisiológico 0,9%, seguida pela aplicação de diversos agentes farmacológicos, como os ácidos graxos essenciais, a colagenase e a sulfadiazina de prata.^(6)^

No Brasil, poucos estudos vêm sendo realizados a respeito das UP e seu tratamento. Os estudos já realizados mostraram que as formas de tratamento variam muito, além de o cumprimento das diretrizes existentes ser, por vezes, inadequado.^(2, 7)^

## OBJETIVO

Avaliar os fatores relacionados ao desenvolvimento da úlcera por pressão em pacientes internados em um hospital de longa permanência, bem como comparar a efetividade de dois tratamentos farmacológicos.

## MÉTODOS

O presente trabalho caracterizou-se como um estudo prospectivo, realizado em pacientes internados em um hospital de longa permanência de Juiz de Fora (MG), que dispõe de 219 leitos. Foram incluídos todos os pacientes portadores de UP (n=14) internados na instituição durante o período avaliado, que foi dede março a maio de 2013, assim como pacientes com risco de desenvolver úlceras (n=16), os quais foram selecionados randomicamente baseando-se na escala de Braden <18. Tal escala é uma ferramenta útil e muito utilizada para avaliar o risco de desenvolvimento da UP, determinado por meio da medida dos parâmetros clínicos de exposição para intensa e prolongada pressão, e da tolerância do tecido a essa pressão. A pontuação na escala de Braden varia de 4 a 23, sendo que pacientes hospitalizados com escore ≥16 pontos são considerados de pequeno risco para desenvolvimento da lesão, enquanto os escores <11 apontam alto risco.

Este estudo foi aprovado pelo Comitê de Ética em Pesquisa da Faculdade de Ciências Médicas e da Saúde de Juiz de Fora (processo 0059/12), e todos os voluntários foram esclarecidos da pesquisa e consentiram na participação por meio da assinatura do Termo de Consentimento Livre e Esclarecido (TCLE).

A coleta dos dados foi realizada partir de registros contidos no prontuário do paciente, com o propósito de registrar as informações sociodemográficas, clínicas, medicamentos sistêmicos, fatores de risco, medidas de prevenção da úlcera e seguimento do tratamento farmacológico e não farmacológico da lesão. Os dados referentes aos exames laboratoriais (hemoglobina, hematócrito, hemácias, leucócitos, neutrófilos, linfócitos, glicose, creatinina, colesterol total e frações e triglicerídeos) foram obtidos a partir de amostras de sangue do paciente em jejum apropriado, coletadas após sua inclusão no estudo. Todos os exames foram realizados conforme rotina do hospital. O tratamento farmacológico da lesão foi escolhido entre sulfadiazina de prata 1% (creme dermatológico) (n=6) e colagenase 0,6UI/g (pomada) (n=8), de acordo com o clínico responsável e sem interferir na rotina da instituição.

Todos os tratamentos foram aplicados uma vez ao dia, no período da manhã, após limpeza da ferida com solução fisiológica por profissional treinado. Durante todo o tratamento, a ferida permaneceu coberta com gaze.

Após o início da avaliação, os pacientes foram acompanhados durante 8 semanas, com intervalos de 15 dias, totalizando 4 avaliações. Em cada uma das avaliações, foram considerados os parâmetros referentes à ferida (dimensões, aspecto e dor), sendo acompanhados e registrados em formulário próprio. As medidas das dimensões das lesões foram feitas considerando-se o comprimento (maior eixo longitudinal, de bordo a bordo do leito da lesão) e largura (menor eixo transversal ao eixo longitudinal, de bordo a bordo do leito da lesão), sendo realizadas com o auxílio de um paquímetro e registradas em centímetros. A área total da ferida foi calculada multiplicando-se o comprimento pela largura (cm^2^). Além dos tratamentos farmacológicos, as medidas não farmacológicas realizadas também foram registradas, para posterior comparação entre os grupos de pacientes.

Os dados coletados foram processados e analisados por meio do programa GraphPad Prism, versão 5.0. Após tabulação, esses dados foram submetidos aos seguintes procedimentos estatísticos: teste exato Fisher para comparação das variáveis nominais como sexo, cor, mobilidade e uso de fralda entre o grupo de pacientes portadores de UP e aqueles sem a ferida; e teste χ^2 ^para comparar os grupos com relação à prevalência das doenças. A diferença entre os grupos em relação ao total de medicamentos em uso foi feito por teste χ^2^, enquanto a comparação entre cada uma das classes de medicamentos foi feita através do teste exato Fisher. Todas as variáveis contínuas foram avaliadas quanto à distribuição normal (teste de D’Agostino e Pearson). Aqueles que apresentaram distribuição normal, os grupos foram comparados por meio da análise de teste *t* não pareado, enquanto os dados que não exibiram distribuição normal foram comparados pelo teste não paramétrico de Mann-Whitney.

Em todas as análises, utilizou-se valor de p<0,05 como estatisticamente significante.

## RESULTADOS

Dentre os 219 leitos disponíveis no hospital de longa permanência na cidade de Juiz de Fora, foram identificados 14 pacientes portadores de UP durante os 3 meses de condução do estudo, demonstrando a prevalência de cerca de 6% para esse tipo de ferida. Dentre tais pacientes, no decorrer das observações, dois deles foram a óbito por sepse e um foi transferido para outra instituição de saúde.

Além dos pacientes portadores da úlcera, pacientes em risco de desenvolver a lesão também foram avaliados, e tais características gerais foram comparadas às daqueles pacientes portadores da lesão (Tabela 1). A comparação entre esses dois grupos exibiu não haver diferença estatística significativa entre ocorrência de úlceras por pressão, segundo idade, sexo, cor da pele, mobilidade e uso de fralda. No entanto, quando comparados os parâmetros hematológicos e bioquímicos (Tabela 2), foram observadas diferenças nos parâmetros hemoglobina, hematócrito e hemácias, sendo que tais valores estavam reduzidos nos pacientes portadores da ferida. Por outro lado, não foram observadas diferenças significativas no que se refere à comparação entre as diferentes classes de medicamentos utilizados pelos grupos (Tabela 3).

**Tabela 1:** Caracterização geral dos pacientes internados em hospital de longa permanência (n=30), portadores de úlcera por pressão ou com risco de desenvolvimento da lesão

Característica	Com úlcera (n=14)	Sem úlcera (n=16)	Valor de p
Idade	63,6±15,4	64,25±17,67	0,92
Sexo			
Masculino	7	9	
Feminino	7	7	1,00
Cor			
Branca	8	5	
Não branca	6	11	0,27
IMC (kg/m^2^)	20,4±2,5	20,7±2,8	0,70
Estado nutricional			
Desnutrição	3	2	
Eutrofia	8	13	
Sobrepeso	0	1	0,47
Mobilidade			
Deambula	3	0	
Não deambula	11	16	0,09
Doenças			
Doenças neurológicas	10	13	
Lesão de MMII	5	3	
Diabetes	2	3	
HAS	7	7	
Infecção urinária	4	0	
Outras	2	6	0,22
Uso de fralda			
Sim	12	15	
Não	2	1	0,60
Uso de colchão “caixa de ovo”			
Sim	8	1	
Não	6	15	-
Número de úlceras por paciente	2 (Mínimo: 1/Máximo: 5)*	-	-

Resultados expressos por média ± desvio padrão.

(-) Não realizada análise estatística; * amplitude de ocorrência de úlceras por pressão por paciente.

IMC: índice de massa corporal; MMII: membros inferiores; HAS: hipertensão arterial sistêmica.

**Tabela 2:** Caracterização bioquímica e hematológica dos pacientes internados em hospital de longa permanência (n=30) portadores de úlcera por pressão ou com risco de desenvolvimento da lesão

Característica	Com úlcera (n=14)	Sem úlcera (n=16)	Valor de p
Hemoglobina (g/dL)	10,5±1,3	12,8±1,5	0,0002*
Hematócrito (%)	32,6±3,9	39,9±4,3	<0,0001*
Hemácia (milhões/mm^3^)	3,9±0,6	4,7±0,7	0,0026*
Leucócito (n/mm^3^)	5.877±1.562	6.694±1.750	0,2009
Neutrófilo (%)	60,4±10,8	67,6±11,7	0,1008
Linfócito (%)	32,5±10,4	26,5±10,0	0,1232
Glicose (mg/dL)	80±13	86±20	0,3984
Creatinina (mg/dL)	0,8±0,4	0,8±0,3	0,8134
Colesterol total (mg/dL)	174±27	207±50	0,0631
LDL (mg/dL)	111±22	138±48	0,1025
HDL (mg/dL)	37±13	36±7	0,6959
Triglicerídeo (mg/dL)	135±76	159±109	0,8330

Resultados expressos por média ± desvio padrão.

* p<0,05 (teste t não pareado).

LDL: lipoproteína de baixa densidade; HDL: lipoproteína de alta densidade.

**Tabela 3:** Prevalência de úlcera por pressão conforme o medicamento sistêmico em uso pelo paciente

Medicamentos	Com úlcera n (%)	Sem úlcera n (%)	Valor de p
Antianêmicos	19 (22)	18 (18)	0,596^3a^
Antibióticos	3 (3)	2 (2)	1,0000^a^
Psicotrópicos	21 (24)	19 (19)	0,4915^a^
Insulina e hipoglicemiantes orais	1 (1)	7 (7)	0,0649^a^
Anti-hipertensivos	9 (10)	9 (9)	1,0000^a^
Analgésicos	4 (5)	4 (4)	1,0000^a^
Diuréticos	6 (7)	10 (10)	0,6133^a^
Outros	23 (27)	30 (30)	0,7542^a^

Total	86 (100)	99 (100)	0,479^1b^

^a^Teste exato de Fisher

^b^Teste χ^2^.

Já nos pacientes portadores de UP, a localização e a classificação das feridas são mostradas na tabela 4, evidenciando o predomínio de úlceras na região sacral, seguidas pelo calcâneo e região do trocânter.

**Tabela 4:** Características das úlceras por pressão em 14 pacientes internados em hospital de longa permanência

Característica	Número de úlceras (n=26) n (%)
Localização	
Sacral	12 (46)
Trocânter	2 (8)
Calcâneo	7 (27)
Outras	5 (19)
Estágio	
I	3 (12)
II	9 (34)
III	11 (42)
IV	3 (12)

A evolução da úlcera em relação ao tratamento (colagenase ou sulfadiazina de prata) foi acompanhada por meio da área da lesão, durante o tempo de avaliação de 8 semanas (Figura 1). Apesar de a maioria dos pacientes ter apresentado uma redução do tamanho médio da ferida, a comparação da área da lesão antes e após o tratamento farmacológico não exibiu diferença estatística. Ainda relacionada à redução da área da lesão, não foi observada diferença significativa entre os dois tratamentos farmacológicos (colagenase ou sulfadiazina de prata; p=0,94).

**Figura 1:** Dimensões das úlceras por pressão (cm2) em 11 pacientes internados em hospital de longa permanência antes a após o tratamento com (A) colagenase (n=8) e com (B) sulfadiazina (n=6) por 8 semanas. Não foram observadas diferenças significativas entre os valores basais e após tratamento no tempo acompanhado (p>0,05)

## DISCUSSÃO

Os dados referentes à prevalência de úlceras por pressão encontrados no hospital investigado no estudo (cerca de 6%) estão de acordo com os da literatura internacional, que demonstram variação entre 3 e 14%, nos Estados Unidos. Já no Brasil, estudos afirmam que a prevalência em hospitais pode chegar a aproximadamente 20%.^(4, 8)^

Além dos pacientes portadores de UP, durante o mesmo período, foi avaliado um grupo de pacientes em risco de desenvolvimento da lesão. Tal comparação exibiu não haver diferença estatística significativa quanto à idade, sexo, mobilidade e uso de fralda. Em relação à cor da pele, também não houve predomínio significativo entre os grupos (p=0,27), ao contrário de observações prévias sugerirem que a pele negra é mais resistente à agressão externa causada pela umidade e fricção, devido a alterações no estrato córneo − que, nos negros, é mais compacto.^(4)^ Adicionalmente, nossos dados não mostraram diferença estatisticamente significativa entre o índice de massa corporal (IMC) e o grau nutricional dos pacientes portadores e não portadores da lesão, ao contrário de achados prévios que demonstram uma relação direta entre IMC e a prevalência de úlcera, sendo que esta aumentava com a desnutrição, devido à perda do “efeito amortecedor” do tecido adiposo.^(9)^ Tal discordância nos resultados pode se dever ao fato de que os dois grupos de pacientes investigados no presente estudo exibiram IMC próximos ao limite inferior da normalidade, sendo a maioria pacientes acamados por longo período em função de suas doenças de base. Além disso, outra possível explicação para a discordância entre os dados apresentados neste estudo e aqueles presentes na literatura pode ser o uso de métodos diferentes para avaliação do estado nutricional do paciente.^(9)^

Por outro lado, com relação aos dados bioquímicos e hematológicos analisados entre pacientes que desenvolveram úlceras por pressão e pacientes sem úlceras, observou-se significativa redução dos valores de hemoglobina (p=0,0002), hematócrito (p<0,0001) e hemácias (p=0,026) nos pacientes portadores da lesão. Sabe-se que diversas doenças podem estar associadas ao desenvolvimento de úlceras por pressão, como *diabetes mellitus*, hipertensão arterial, doenças neurológicas e infecções, porém apenas a anemia mostrou relação significativa em nosso estudo.^(9)^ A deficiência na série vermelha é comum em pacientes com úlceras por pressão, não só devido ao estado nutricional deficiente, mas também pelos efeitos das citocinas inflamatórias, decorrentes da lesão, nas células progenitoras eritroides.^(10)^ Essas condições são limitantes a mobilidade do paciente e prejudicam a irrigação sanguínea, reduzindo o transporte de oxigênio e de nutrientes à ferida.^(9)^

Em contrapartida, não foi observada associação estatisticamente significante entre os valores de leucócitos totais (p=0,2009) e o desenvolvimento de úlcera de por pressã, quando comparado os dois grupos observados, assim como o valor de neutrófilos (p=0,1008). De acordo com a literatura, o aumento de neutrófilos parece indicar um mau prognóstico das úlceras por pressão, devido a mediadores inflamatórios desencadeados pelas lesões, elevando também os leucócitos totais.^(9)^ No entanto, no presente trabalho, essa relação não foi observada.

Ao se avaliarem os medicamentos em uso pelos pacientes internados, houve grande diversificação das classes, com predominância de psicotrópicos, seguidos por antianêmicos e anti-hipertensivos, não sendo apresentada diferença estatística significativa na utilização pelos pacientes nos grupos de portadores e não portadores da lesão (p=0,4791). Quanto aos medicamentos utilizados, sabe-se que principalmente aqueles de uso contínuo, embora necessários, podem contribuir para o desenvolvimento de úlcera, como os agentes hipotensores, que podem alterar o fluxo sanguíneo, reduzindo a perfusão dos tecidos e acarretando diminuição da tolerância cutânea à pressão.^(4)^

Em relação à localização das úlceras por pressão, os dados encontrados conferem com a descrição da literatura, apresentando um predomínio de úlceras na região sacral, seguidas pelo calcâneo e região do trocânter.^(4, 11)-15)^ Tal fato se explica pelo posicionamento dorsal horizontal, que potencializa as forças de fricção e cisalhamento nos pacientes, os quais permanecem por muito tempo nessa mesma posição.^(11)^

A comparação da área da ferida entre o início e o final do tempo de observação de 8 semanas demonstra não existir diferença significativa quanto à redução após ambos os tratamentos. No entanto, esses valores ficaram bem próximos ao limite estabelecido de p=0,05, sendo que, no tratamento com colagenase, essa comparação rendeu um valor de p=0,06 e, com sulfadiazina de prata, o valor de p foi de 0,07 (excluído dessa última análise um paciente que apresentou complicações clínicas em função de uma infecção urinária recorrente, o que, provavelmente, explica o crescimento da área da ferida sacral após tratamento). Além disso, a comparação entre os dois tratamentos farmacológicos não demonstrou diferença quanto à redução da área da ferida durante as 8 semanas de observação.

Apesar dos interessantes achados, o número reduzido de pacientes e o curto tempo de observação dos tratamentos farmacológicos (8 semanas) foram importantes limitações ao estudo.

## CONCLUSÃO

A prevalência de úlcera por pressão no hospital de longa permanência de Juiz de Fora evidencia concordância com os dados nacionais e internacionais sobre o tema.

Adicionalmente, observou-se relação estatisticamente significativa entre desenvolvimento de úlcera por pressão e valores baixos de hemoglobina, hematócrito e hemácias, podendo tais parâmetros serem úteis na identificação dos pacientes em risco de desenvolver a lesão, além do monitoramento e tratamento de pacientes portadores dessa ferida. Nesse sentido, tais achados, somados às medidas já conhecidas para prevenção da úlcera por pressão, tornam-se informações de extrema relevância a serem amplamente divulgadas e incorporadas na rotina dos profissionais da saúde que trabalham com pacientes em risco de desenvolvimento da ferida.

Ao final das avaliações, a comparação entre os tratamentos das úlceras por pressão com colagenase e sulfadiazina de prata demonstrou não haver diferença estatística significativa na diminuição da área da ferida entre os tratamentos, embora tanto a colagenase como a sulfadiazina tenham evidenciado redução média da lesão.
